# Notes on Preparations for the Sick

**Published:** 1903-09

**Authors:** 


### Notes on Preparations for the Sick.

**Manganese Citrate; Manganese and Iron Citrate; Manganese and Iron Phosphate.**—Burroughs, Wellcome & Co., London.—Manganese has long had a reputation in the treatment of debilitated conditions associated with anæmia, but the preparation known as manganese dioxide, in the insoluble form, has been the only one available. The above preparations are all in the form of scales, which are freely soluble in water. The citrate contains 12 per cent. of manganese, and the other preparations contain 7 per cent. in combination with 14 per cent. of iron. They are also prepared in tabloid forms, each having three or five grains.

**Tabloid:** Quinine Salicylate, gr. j.—This drug is also prepared in a tabloid dose of three grains. Their convenience and value are obvious.

**Soloid:** Romanowsky stain (Leishman's powder).—These soloids have been found to answer their purpose well, and are always ready for use when required.

**Purgen** is a new synthetically prepared purgative, having the composition indicated by the compound name of dihydroxy-phthalophenone. The discovery of the action of this substance is due to the conjoint work of chemists and pharmacologists. For a long time the attention of pharmacological chemists has been directed to the chemical nature of the purgative principles of the naturally occurring aperients such as aloes, rhubarb, etc. These researches culminated in the discovery that the essential purgative principle was glucosidal in nature, and that the purgative glucosides, although differing slightly *inter se*, all yielded, upon appropriate chemical treatment, an anthraquinone derivative, and that further this anthraquinone derivative when administered to men produced a purgative effect. A number of these anthraquinone derivatives have been tested pharmacologically, and certainly possess, as compared with the older purgatives, all those advantages appertaining to a pure chemical substance in contradistinction to a crude mixture of inconstant composition.

Purgen has a pleasant taste, is constant in action, it causes painless evacuation of the bowels, and is only very slightly absorbed; it therefore gives rise to no secondary irritative effects, and does not change the colour of the urine. It is prepared in tablets of different doses from gr. $\frac{3}{4}$ to gr. viiss. The agents for this country are Messrs. Kirby & Co., London.

**Laxoin.**—This is the same chemical compound prepared in palatinoid form (gr. ij.), No. 200, by Messrs. Oppenheimer, Son & Co., London.

**Selama Water.**—142 Gray's Inn Road, London.—In Northern Africa, close to the Mediterranean shore, the famous Selama Artesian spring was discovered accidentally a short time ago. When boring for petroleum the tool struck a powerful water vein at a depth of 900 feet. Highly mineralised, warm and beautifully clear, the carbonic acid gas mixed with the water causes it to rise in a sparkling jet to a varying height of 12 to 50 feet above the surface ground.

This water, when oxygenised, is called Icilma, and is used in the manufacture of Selama Soaps, and of Icilma Fluor Cream. These preparations are believed to have a remarkably sedative effect in all conditions of irritation of the skin.

An analysis by Mr. Oliver Davis, B.Sc., shows that the water contains calcium carbonate ($Ca\,Co_3$) dissolved by carbon dioxide ($Co_2$). It contains also magnesium and calcium sulphate, with sodium chloride, and traces of iron and alumina.

If all which is said of this water is verified by further experience, we shall have at our command another potent Bethesda, which can be brought within the reach of all who need healing waters.

**"Velvril" Film Dressing.**—THOS. CHRISTY & Co., London, E.C.—This consists of a yellow flexible material applied over a wound which has been washed with acetone and wetted with "velvril" solution. The advantage claimed is that it enables the wound to be looked at through the film. As it is desirable, however, to apply an aseptic dressing over the velvril, and as we practically never want to look at a wound until it is necessary to change the dressing, the usefulness of the material seems doubtful, in the larger wounds at all events.

For smaller wounds—to take the place of collodion—where no dressings are required, it has perhaps a greater sphere of usefulness, for it sticks to the wound closely and well.

It might also take the place of oiled silk as a protective agent without being tightly fixed to the wound.

**Mennen's Borated Talcum Toilet Powder.**—11 Queen Victoria Street, London.—This is a sterilised powder, composed of magnesium silicate and boracic acid. It appears to be a soft and agreeable powder, possessing all the qualities necessary for toilet purposes, both for infants and for adults afflicted with abnormal irritation from any external causes. It is also an excellent tooth powder. Analysis (Oliver C. M. Davis, B.Sc.) shows it to be a fine powder, delicately perfumed, consisting of magnesium silicate and boracic acid, with no starch or any other deleterious ingredient.

**Isarol.**—REBMAN, Limited, London.—Is a dark brown viscous liquid of aromatic odour, soluble in water, and easily miscible with fats. It appears to have qualities resembling those of Ichthyol, and is obtained from certain bituminous Alpine schists.

Professor Eggar's report states that Isarol is absolutely identical therapeutically with Ichthyol, and chemically it is the ammonium sulphoichthyolicum of the Swiss pharmocopeia.

**Maltico**: an infants' food prepared from milk and extracts of various nutritive malted cereals. — TAYLOR & SONS, Portsmouth.—It has been found to contain a large amount of proteid nourishment, with maltose, dextrine, and fat. Its ash forms a milk-like emulsion with water, and contains phosphates. Mr. Oliver Davis's analysis shows that the food does not contain any starch. It should be therefore a safe and valuable food for early life.

